# Best PEEP trials are dependent on tidal volume

**DOI:** 10.1186/s13054-018-2047-4

**Published:** 2018-05-02

**Authors:** Andrew C. McKown, Matthew W. Semler, Todd W. Rice

**Affiliations:** Division of Allergy, Pulmonary, and Critical Care Medicine, Department of Medicine, Vanderbilt University Medical Center, T-1218 MCN, 1161 21st Ave S., Nashville, TN 37232-2650 USA

## Abstract

**Abstract:**

Determining the optimal positive end-expiratory pressure (PEEP) in patients with acute respiratory distress syndrome remains an area of active investigation. Most trials individualizing PEEP optimize one physiologic parameter (e.g., driving pressure) by titrating PEEP while holding other ventilator settings constant. Optimal PEEP, however, may depend on the tidal volume, and changing the tidal volume with which a best PEEP trial is performed may lead to different best PEEP settings in the same patient.

**Trial registration:**

ClinicalTrials.gov, NCT02871102. Registered on 12 August 2016.

Positive end-expiratory pressure (PEEP) may mitigate ventilator-induced lung injury in acute respiratory distress syndrome (ARDS) by recruiting collapsed alveolar units, thereby reducing stress raisers and minimizing atelectrauma [1]. Excessive PEEP, however, may cause barotrauma and biotrauma from alveolar hyperdistension. Many studies have attempted to identify the best PEEP for individual patients [2], including the recent Alveolar Recruitment for ARDS Trial (ART) which reported a higher mortality with use of a recruitment maneuver and titrated PEEP compared with use of lower PEEP [3].

We hypothesized that best PEEP, as selected by respiratory system compliance (C_RS_) or driving pressure (end-inspiratory plateau pressure minus PEEP), is contingent upon the tidal volume (V_T_) delivered. Figures 1 and 2 display data from one patient enrolled in our trial (ClinicalTrials.gov NCT02871102), which uses a recruitment maneuver and decremental PEEP protocol similar to the ART trial, but with multiple V_T_ tested for 2 minutes each at every PEEP level [3]. The curves demonstrate that selection of PEEP by point of maximal compliance or minimal driving pressure varies substantially for a single patient depending on the V_T_ with which the best PEEP trial is conducted. This likely occurs due to tidal recruitment.

**Fig. 1:**
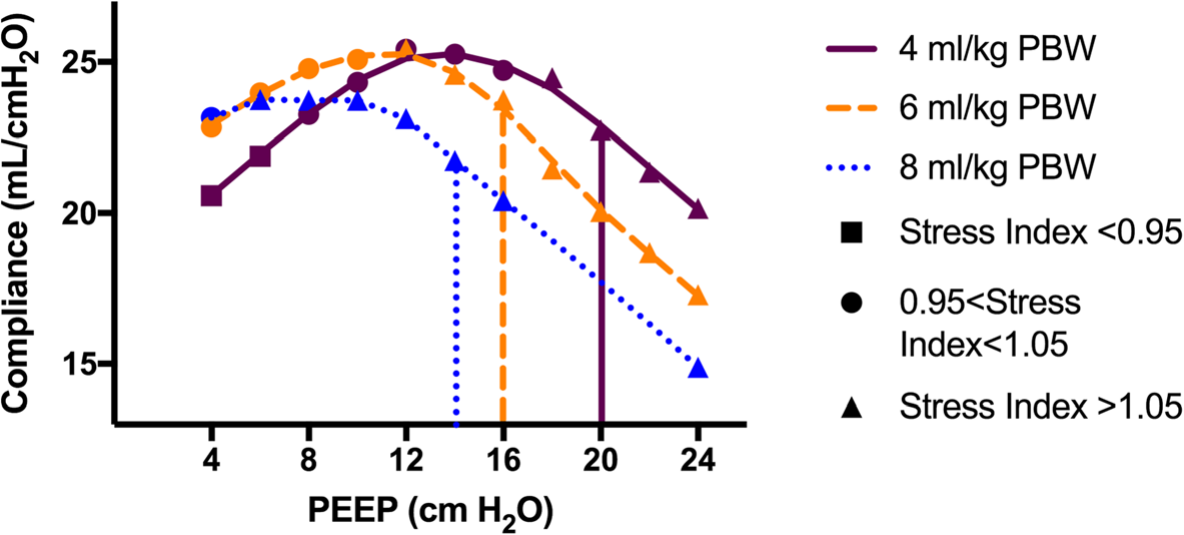
Point of maximal compliance depends on tidal volume used in PEEP trial. Plotted markers represent static compliance of respiratory system (C_RS_) at each PEEP level during a decremental PEEP trial for a single patient. Marker shapes correspond to measured stress index for the PEEP–tidal volume pairing, and marker color corresponds to tidal volume. Loess curve connects points with identical tidal volumes. Vertical lines indicate the PEEP selected by ART protocol for a given tidal volume. PBW predicted body weight, PEEP positive end-expiratory pressure

**Fig. 2:**
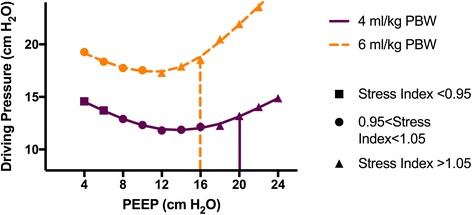
Point of minimal driving pressure depends on tidal volume used in PEEP trial. Plotted markers are measured driving pressure at each PEEP level for a given tidal volume. Marker shapes coded by stress index, and superimposed loess lines show estimated driving pressure over the range of PEEP by tidal volume. Corresponding vertical lines indicate the PEEP selected by ART protocol for a given tidal volume. PBW predicted body weight, PEEP positive end-expiratory pressure

Our findings suggest one possible mechanism for the ART results. The ART intervention utilized a decremental PEEP trial at a V_T_ of 5 ml/kg predicted body weight (PBW), and then set PEEP at 2 cmH_2_O above the PEEP level found to have the maximal C_RS_. If multiple PEEP levels had C_RS_ measures within 1 ml/cmH_2_O, the highest PEEP level was chosen. This protocol likely resulted in the use of PEEP levels in the intervention arm associated with a stress index of > 1.05, suggesting the presence of tidal hyperinflation [4], as demonstrated in our patient by the vertical lines in Fig. 1. Moreover, the curves predict an even greater degree of tidal hyperinflation for patients whose highest PEEP based on maximal compliance was set at one V_T_, but who then received a higher V_T_ for clinical management. Indeed, in the ART trial, intervention patients had a day 1 mean V_T_ of 5.6 ml/kg PBW, implying that many received a V_T_ above that used to select the optimum PEEP.

How to individualize PEEP for patients with ARDS remains a conundrum. Our finding is important because it implies that carefully titrated PEEP may not apply outside of the ventilator parameters with which PEEP was tested and that changes in tidal volume likely influence optimal PEEP.
